# The Efficacy and Safety of Dual-Frequency Ultrasound for Improving Skin Hydration and Erythema in Patients with Rosacea and Acne

**DOI:** 10.3390/jcm10040834

**Published:** 2021-02-18

**Authors:** Young Jae Kim, Ik Jun Moon, Hae Woong Lee, Chong Hyun Won, Sung Eun Chang, Mi Woo Lee, Jee Ho Choi, Woo Jin Lee

**Affiliations:** 1 Asan Medical Center, Department of Dermatology, University of Ulsan College of Medicine, Seoul 05505, Korea; assayoungjae@naver.com (Y.J.K.); ikjun.moon@gmail.com (I.J.M.); chwon98@chol.com (C.H.W.); changse2016@gmail.com (S.E.C.); miumiu@amc.seoul.kr (M.W.L.); jhchoy@amc.seoul.kr (J.H.C.); 2 Louis Dermatology Clinic, Guri-si 11948, Gyeonggi-do, Korea; louiskin@naver.com

**Keywords:** ultrasound, facial erythema, inflammation, skin, rosacea, acne

## Abstract

Inflammatory skin diseases, such as rosacea and acne, are major causes of facial erythema and accompanying skin barrier dysfunction. Several methods to restore the impaired skin barrier and improve facial erythema, such as medication, radiofrequency, laser, and ultrasound therapy were attempted. This study evaluated the efficacy and safety of dual-frequency ultrasound with impulse mode, for improving skin hydration and erythema in Asian subjects with rosacea and acne. Twenty-six subjects with facial erythema received an ultrasound treatment once per week, for 4 weeks, over both cheeks. The erythema index and transepidermal water loss (TEWL) were measured at each visit. Clinicians assessed the erythema improvement and patients evaluated their satisfaction level. The average decrease in TEWL and erythema index at 6 weeks was 5.37 ± 13.22 g·h^−1^·m^−2^ (*p* = 0.020) and 39.73 ± 44.21 (*p* = 0.010), respectively. The clinician’s erythema assessment and the subject satisfaction questionnaire score significantly improved at final follow-up (*p* < 0.001; *p* = 0.003, respectively). No serious adverse effects were observed during the treatment and follow-up periods. The dual-frequency ultrasound with impulse mode appears to be effective and safe for improving skin hydration and erythema in patients with rosacea and acne.

## 1. Introduction

Facial erythema is caused by a variety of factors that induce cutaneous blood vessel dilatation and increase the blood flow to the skin. Primary skin diseases, such as rosacea and acne, are representative inflammatory skin conditions that cause facial erythema. Persistent facial erythema was found in 87% of patients with rosacea [1] and 66% of patients with acne treated with isotretinoin [2]. Matrix metalloproteinases (MMPs) regulates the inflammatory process that stimulates vascular permeability. The increased levels of the MMPs attribute to skin barrier disruption and inflammation in both diseases [3,4,5].

Ultrasound is used for skin care and antiaging treatment, due to its ability to restore the skin barrier by inhibiting the MMPs [3,4,5]. A recent ultrasound device called SONO STYLER (WEYERGANS^®^, Dueren, Germany) is differentiated from conventional ultrasound devices, in that, it utilizes a specialized impulse mode with a constant wave period. This regular interval energy transfer enables a more accurate energy delivery, intensifying it over the targeted tissue, without unnecessary thermal damage. Until now, few reports investigated the efficacy and safety of ultrasound for the treatment of facial erythema. However, there is no study with this novel dual-frequency ultrasound device, SONO STYLER. The present study evaluated the efficacy and safety of an impulse mode dual-frequency ultrasound with a frequency of 1/3 MHz, for improving skin hydration and erythema in patients with rosacea and acne.

## 2. Materials and Methods

### 2.1. Subjects

Men and women aged over 18 years with facial erythema related with rosacea and acne were included in this study. A total of 28 Asian patients (19 females, 9 males) were enrolled. Exclusion criteria were as follows—history of anti-inflammatory medication use, such as antibiotics and isotretinoin, within the last 3 months; history of facial surgical or laser treatments; cutaneous infectious disease or inflammatory diseases except rosacea and acne; and any other systemic disease. Patients with over five discrete inflammatory nodules and papules were also excluded. In addition, patients with implanted devices, including pacemakers, defibrillators, and prosthetic metal implants, were also excluded. As a result, 26 patients (17 females and 9 males) completed the treatment.

### 2.2. Treatment Protocol

The patients received a dual-frequency ultrasound treatment using the impulse mode once per week for 4 weeks, over both cheeks. The patients were laid in a supine position, and the US applicator was gently rotated for 5 min at each cheek. A power of 1.0 W/cm^2^ with a dual frequency of 1/3 MHz was utilized, which is a basic parameter of the impulse mode of the SONO STYLER device, to focus the energy to the superficial area of the skin and minimize thermal damage.

### 2.3. Efficacy Evaluation

All patients were followed up at each treatment, once a week, and two weeks after the last treatment (baseline and weeks 2, 4, and 6). Standardized digital clinical photographs were taken (Front and bilateral oblique views at 45 degrees, Nikon Coolpix 4500; Nikon, Tokyo, Japan) and clinician’s erythema assessment (CEA) scores and Subject satisfaction questionnaire (SSQ) scores were obtained at each follow-up. Transepidermal water loss (TEWL) and skin color change were recorded at every visit. These were measured at both cheeks, and the average values were calculated. The TEWL (described in g·h^−1^·m^−2^, Cutometer^®^; Courage-Khazaka Electronic, Köln, Germany) was measured on the most erythematous lesion on each cheek and the average value of both cheeks was recorded. The skin color changes of thirteen patients were assessed by another tool (described in R, Mexameter^®^; a probe of Cutometer^®^).

All biological indices were measured by one dermatologist. CEA scores were obtained at each visit using a 5-point scale (0: complete response; 1: almost complete response; 2: partial response with mild erythema; 3: partial response with moderate erythema; and 4: no or little response with severe erythema). SSQ scores for treatment response were assessed at each visit by a 4-point scale questionnaire (1: very satisfactory; 2: satisfactory; 3: little satisfactory; or 4: unsatisfactory).

### 2.4. Safety Evaluation

With regard to safety issues, any adverse events occurring during the entire study period were recorded. Unexpectedly worsened erythema, pruritus, and a burning sensation were all monitored as safety parameters during the ultrasound treatment. The degree of adverse effects was considered before deciding to continue the ultrasound treatment.

### 2.5. Statistical Analysis

Statistical analyses were performed using the SPSS software, version 19.0 for Windows (SPSS Inc., Chicago, IL, USA). Statistical comparisons of pre- and post-treatment variables were performed using the paired *t*-test and Wilcoxon signed rank test. *p*-values < 0.05 were considered to be statistically significant.

## 3. Results

**Figure 1:**
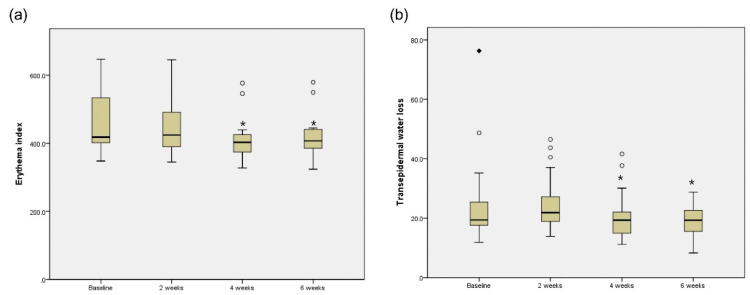
Erythema index (**a**) and TEWL (**b**) at baseline, 2 weeks, 4 weeks, and 6 weeks after treatment with dual-frequency ultrasound. The boxes show the interquartile range and the bars inside the box represent the median value. The outside rhombus and circles indicate the lowest and highest data. * *p* < 0.05.

Twenty-eight (19 females and 9 males) Asian patients were initially enrolled in the present study. Of those, 26 (17 females and 9 males) completed the treatment. The mean patient age was 37.04 ± 10.44 years (range, 23 to 55 years). Among 26 patients, 15 patients revealed facial erythema predominantly related with rosacea and the other 11 patients showed acne predominance (Table 1). The mean TEWL values decreased from the baseline 28.91 to 18.82 g·h^−1^·m^−2^ at 6 weeks. The average decrease in TEWL at 6 weeks (two weeks after treatment completion) was 5.37 ± 13.22 g·h^−1^·m^−2^ (*p* = 0.020). The decreased tendency of TEWL was statistically significant after 4 weeks of follow-up (*p* = 0.033). The decrease in the mean value of skin color changes was statistically significant, from 461.80 at baseline to 422.07 at 6 weeks (*p* = 0.010). The decrease in the erythema index was statistically significant after 4 weeks of follow up (*p* = 0.023) (Figure 1). When the decrease of erythema index was expressed as a relative percentage value, it was rated as 2.82% at 2 weeks, 9.62% at 4 weeks, and 8.60% at 6 weeks follow-up.

**Figure 2:**
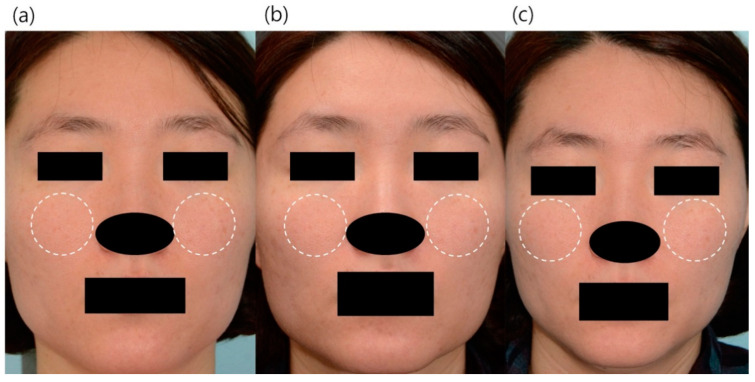
Clinical digital photography of the representative subjects. (**a**) 35-year-old female, baseline TEWL: 541, baseline erythema index: 25.05 (**b**) TEWL at 2 weeks post treatment was 506 g·h^−1^·m^−2^ (6.5% decreased from baseline) and erythema index at 2 weeks post treatment was 23.90 (4.2% decreased from baseline). (**c**) TEWL at 6 weeks post treatment was 441 (18.5% decreased from baseline) and erythema index at 6 weeks post treatment was 20.65 (17.6% decreased from baseline). The white dotted circles were the most erythematous parts where the severity of erythema was measured, and they generally coincided with the parts where the patient expressed satisfaction with improvement in erythema.

**Figure 3:**
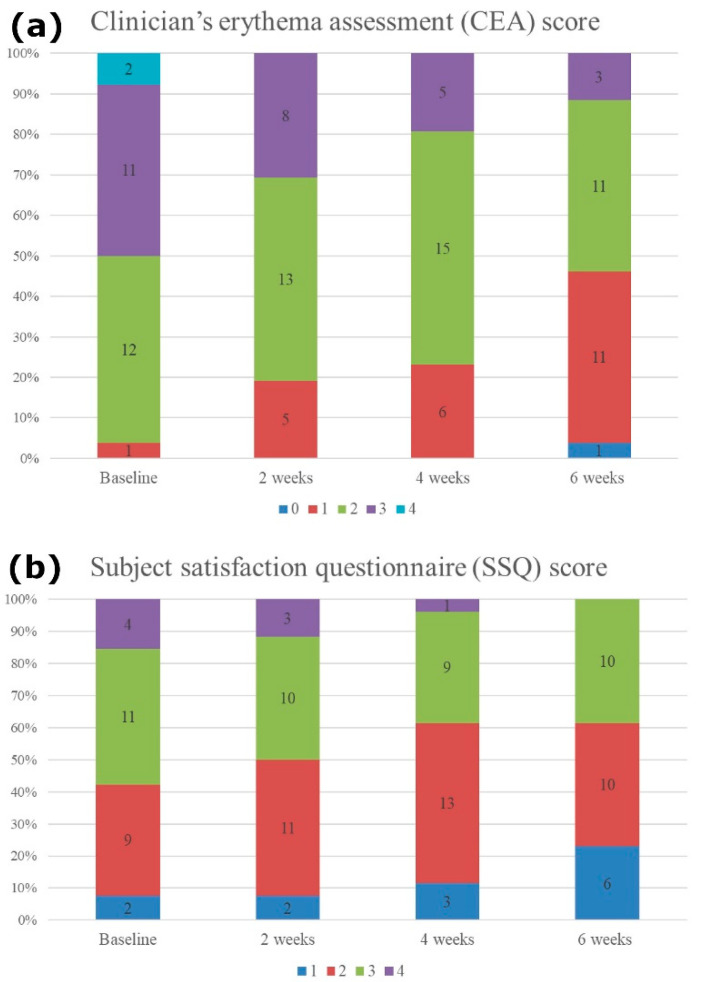
(**a**) Clinician’s erythema assessment (CEA) score and (**b**) subject satisfaction questionnaire (SSQ) score. CEA value was significantly decreased from the baseline 2.53 to 1.61 at 6 weeks follow-up (*p* < 0.001). The SSQ scores also significantly decreased from the baseline 2.65 to 2.15 at 6 weeks follow-up (*p* = 0.003).

The clinical improvement of the facial erythema was monitored using weekly standardized digital clinical photographs of the anterior and 45° oblique lateral views (Figure 2). All patients showed reduced erythema, regardless of the initial erythema severity. When assessed by an investigator using the CEA score, the score was significantly decreased from the baseline score of 2.53 at first treatment to 1.61 at final treatment (*p* < 0.001). The SSQ scores for treatment response also significantly decreased from 2.65 at baseline to 2.15 at 6 weeks post treatment. (*p* = 0.003) (Figure 3). During the whole treatment and follow-up period, no safety issues related to the ultrasound device were observed.

## 4. Discussion

Ultrasound was broadly applied to the treatment of various skin conditions, including cutaneous wounds, inflammatory skin disorders, and esthetic problems [6,7]. Moreover, an improvement in skin laxity, body contouring through fat reduction, and skin anti-aging effects with bio-revitalization were newly reported benefits of the ultrasound treatment [8,9]. Although previous studies supported the skin care abilities of ultrasound [3,4,5], the underlying mechanisms of this novel therapy should be more clearly elucidated, because it is a relatively new treatment modality. The advancement of ultrasound devices is steadily growing, raising concerns about the applicability and efficacy of the newly developed devices. SONO STYLER is one such recently developed device, with a dual-frequency ultrasound stacking energy, with an impulse mode of regular intermittent wave period. This novel technology is designed to maintain the optimal energy level by minimizing the effect of incorrect wave interference. In contrast to the conventional ultrasound devices, it delivers more fine-tuned energy to the target tissue without excessive thermal damage.

Facial erythema is one of the reported indications for ultrasound treatment. Park et al. reported that ultrasound with frequencies of 3/4.5 MHz at an intensity of 2.0 W/cm^2^ (Hyperlux; M.I.Tech Co., Ltd., Daejeon, Korea) was effective for the treatment of facial erythema [3]. However, there is no study exploring the efficacy of SONO STYLER, a dual-frequency ultrasound device with frequencies of 1/3 MHz, at an intensity of 1.0 W/cm^2^. In this study, we prospectively evaluated the efficacy and safety of SONO STYLER in the treatment of facial erythema and revealed its effectiveness and safety. We found significant decrease in the TEWL and erythema index after treatment, implying skin barrier restoration and anti-inflammatory action of the ultrasound.

In rosacea and acne, skin barrier disruption and inflammatory skin environment with an increased level of MMPs were reported [10,11,12]. For example, MMP-9 in the skin of patients with rosacea might increase the levels of kallikrein 5 and LL-37, which are the key molecules in the pathophysiology of rosacea [13]. Moreover, the stimulated MMP-9 in *P. acne* (recently renamed as *C. acne*) induced greater inflammation in the skin [14]. It was reported that the overexpressed MMPs were suppressed by the application of ultrasound in vitro [15]. Few clinical studies practically supported this anti-inflammatory effect of the ultrasound, particularly in patients with rosacea and acne [5,12,15]. The disturbed skin barrier could also be restored by the ultrasound. A potential underlying principle for the recovery of skin barrier is the epidermal calcium gradient change. Ultrasound can induce skin barrier recovery by changing the epidermal calcium gradient, which provokes further lamellar body secretion and lipid synthesis [16]. Since the increased lamellar body secretion facilitates secondary cytokine synthesis and release, the barrier recovery can be accelerated. Furthermore, when ultrasound transfers energy to the epidermis, the spaces between the keratinocytes are enlarged and microcavities are produced by acoustic mechanical stress, thereby increasing the permeability of the epidermis. This so called sonophoresis cleans the microenvironment of the epidermis, ultimately reducing the inflammatory debris and upregulating the epidermal recovery [17].

We evaluated the clinical efficacy and safety of the novel dual-frequency ultrasound device with impulse mode, for facial erythema. Based on the results of this study, the dual-frequency ultrasound effectively improved the facial erythema and skin hydration. The ultrasound in this study was applied with lower frequencies and a lower intensity than those reported in previous studies. Despite the lower energy levels, the effectiveness was not inferior, thus minimizing the risk of adverse effects. Subject satisfaction was also significantly improved. Another interesting point is that, unlike those in previously reported studies [1,2], the subjects in this study did not use any anti-inflammatory agents. That is, this study demonstrated the efficacy of the dual-frequency ultrasound in facial erythema treatment, by excluding the potential influence of medications. Most patients with facial erythema due to rosacea reported an improvement in the burning and stinging sensation, as well as that of erythema, with increased subject satisfaction. These results suggest that the disrupted skin barrier and inflammatory conditions of the skin could be improved by using an ultrasound without medications.

In addition, this study revealed a prominent improvement of the facial erythema, irrespective of its initial severity. Although the number of patients with each subtype of rosacea was small, the skin hydration and erythema were significantly improved in all patients, after the SONO STYLER treatments. Since the ultrasound has the effect of sonophoresis, increasing the permeability of the epidermal barrier, a synergistic effect using various combinations with topical anti-erythema medication can be achieved.

With regards to safety, none of the patients complained of discomfort, pain, or a stinging sensation, during the entire treatment. An aggravation of the facial erythema after the procedure was not observed in any patient, resulting in the absence of a downtime of the ultrasound device. This characteristic of the ultrasound device would be a great advantage in the application to the weekly skin medical care of patients, with facial erythema, especially in patients with sensitive skin. However, this study bears inherent limitations in the small sample size and uncontrolled confounding factors related to the erythema, such as variations in lifestyles, emotional stress, exercise, and diet. Since this study was initially designed as a clinical prospective pilot study, control groups were not included. Further a randomized, blinded, placebo-controlled study with a large sample size would be helpful to prove the effect of a dual-frequency ultrasound device with a higher level of evidence.

## 5. Conclusions

The dual-frequency ultrasound with an impulse mode was safe and effective for improving facial erythema related to rosacea and acne. Additional clinical studies in combination with anti-inflammatory medications would be helpful to establish the best treatment protocol for facial erythema, with this novel ultrasound device.

## Figures and Tables

**Table 1:** Patients’ demographics and summary of clinical results.

Characteristics	Facial Erythema Mainly with Acne	Facial Erythema Mainly with Rosacea	Total
Total patients, *n*	11	15 (ETR:10, PPR:3, Mixed:2)	26
Age, y (mean ± SD)	28.6 ± 6.2	43.2 ± 8.4	37.0 ± 10.4
Sex ratio (male : female)	6 : 5	3 : 12	9 : 17
Baseline erythema index	523.0 ± 147.8	450.7 ± 91.7	461.8 ± 97.8
Erythema index at 6 weeks follow-up	493.5 ± 121.6 (*p* = 0.18)	409.1 ± 59.3 (*p* = 0.023)	422.1 ± 71.9 (*p* = 0.010)
Baseline TEWL index	20.8 ± 6.3	26.9 ± 16.7	24.3 ± 13.5
TEWL index at 6 weeks follow-up	18.6 ± 6.0 (*p* = 0.290)	19.2 ± 5.0 (*p* = 0.036)	18.9 ± 5.3 (*p* = 0.020)
Baseline CEA score	2.72 ± 0.90	2.40 ± 0.51	2.53 ± 0.71
CEA score at 6 weeks follow-up	1.54 ± 0.93 (*p* = 0.023)	1.67 ± 0.62 (*p* = 0.002)	1.61 ± 0.75 (*p* < 0.001)
Baseline SSQ score	2.82 ± 0.87	2.53 ± 0.83	2.65 ± 0.84
SSQ score at 6 weeks follow-up	2.27 ± 0.78 (*p* = 0.058)	2.06 ± 0.79 (*p* = 0.020)	2.15 ± 0.78 (*p* = 0.003)

ETR: erythematotelangiectatic rosacea; PPR: papulopustular rosacea; TEWL: transepidermal water loss; CEA: clinician’s erythema assessment scores; and SSQ: subject satisfaction questionnaire scores.
